# DATA FOR ACTION: CDC’S BEHAVIORAL RISK FACTOR SURVEILLANCE SYSTEM (BRFSS) COGNITIVE DECLINE AND CAREGIVING DATA

**DOI:** 10.1093/geroni/igz038.1339

**Published:** 2019-11-08

**Authors:** Lisa C McGuire

**Affiliations:** CDC, Atlanta, Georgia, United States

## Abstract

The Centers for Disease Control and Prevention’s (CDC) Behavioral Risk Factor Surveillance System (BRFSS) is the world’s largest ongoing health survey, administered in all 50 U.S. states, as well as the District of Columbia and the three U.S. territories, with data collected from more than 400,000 respondents. The BRFSS collects data on cognitive decline and caregiving as well as many health behaviors, annually. In 2015-2017, the 6-item cognitive decline module has been administered in 49 states, DC, and Puerto Rico, while the 9-item caregiving has been administered in 44 states, DC, and Puerto Rico. CDC’s Alzheimer’s Disease and Healthy Aging Program has developed many data for action resources for use by states and other partners to help identify populations and communities most at need. This presentation will describe the series of resources to facilitate data utilization among Tribal Communities, including state and race/ethnicity-specific infographics, topic-specific briefs, and the online portal.

